# Regulation of Constitutive Neutrophil Apoptosis Due to House Dust Mite Allergen in Normal and Allergic Rhinitis Subjects

**DOI:** 10.1371/journal.pone.0105814

**Published:** 2014-09-22

**Authors:** Eun Hye Kim, Ji-Sook Lee, Na Rae Lee, Seung Yeop Baek, Eun Jeong Kim, Soo Jin Lee, In Sik Kim

**Affiliations:** 1 Department of Biomedical Laboratory Science, School of Medicine, Eulji University, Daejeon, Republic of Korea; 2 Department of Clinical Laboratory Science, Wonkwang Health Science University, Iksan, Republic of Korea; 3 Department of Senior Healthcare, BK21 plus program, Graduated School, Eulji University, Daejeon, Republic of Korea; 4 Department of Pediatrics, School of Medicine, Eulji University, Daejeon, Republic of Korea; Rutgers - New Jersey Medical School, United States of America

## Abstract

House dust mite (HDM) is a primary allergen in allergic rhinitis (AR) and asthma. Neutrophil apoptosis is associated with allergic diseases and innate immunity to infection. The present study examined how HDM affects constitutive neutrophil apoptosis in normal and AR subjects. Total IgE increased in AR subjects when compared to normal subjects, and patients with AR were HDM-specific IgE positive (+), which is specific IgE to *Dermatophagoides pteronissinus* and *Dermatophagoides farinae*. In normal and AR subjects, neutrophil apoptosis was inhibited by extract of *Dermatophagoides pteronissinus* (DP), but not by extract of *Dermatophagoides farina* (DF). Aprotinin (serine protease inhibitor) and E64 (cysteine protease inhibitor) have no effect on neutrophil apoptosis due to DP. The anti-apoptotic effect of DP was blocked by TLR4i, an inhibitor of TLR4, rottlerin, an inhibitor of PKCδ, PD98059, an inhibitor of ERK, and BAY-11-7085, an inhibitor of NF-κB. DP induced PKCδ, ERK, and NF-κB activation in a time-dependent manner. DP inhibited the cleavage of procaspase 3 and procaspase 9. The expression of IL-6, IL-8, TNF-α, G-CSF, GM-CSF, and CCL2 increased in the supernatant collected from the normal and AR neutrophils after DP treatment and the supernatant inhibited the apoptosis of normal and AR neutrophils. In summary, DP has anti-apoptotic effects on neutrophils of normal and AR subjects through the TLR4/PKCδ/ERK/NF-κB pathway, and this finding may contribute to solution of the pathogenic mechanism of allergic diseases triggered by DP.

## Introduction

Rhinitis is classified as allergic or non-allergic based on allergen sensitization [1], [2]. Allergic rhinitis (AR) is characterized by rhinorrhea, stuffy nose due to nasal obstruction, sneezing and itching, and its symptoms are caused by an immunological mechanism after exposure to allergen. AR is deeply related to other atopic diseases such as asthma. House dust mite (HDM), which includes two main species, *Dermatophagoides pteronissinus* and *Dermatophagoides farinae*, plays an important role in the onset and aggravation of allergic diseases, including AR [3]–[5]. Total IgE in the serum of AR subjects is high relative to normal subjects and HDM-specific IgE (HDM IgE) also appears in AR patients. A recent study demonstrated the importance of immune dysregulation by HDM in AR [6].

Neutrophils are an abundant leukocyte in the peripheral blood that function as the first line of defense against infection [7], [8]. Neutrophils have a short half-life of 6 to 18 hrs in circulation, but their survival time increases after migrating from circulation into sites of inflammation. This increase in survival time is triggered by inhibition of apoptosis by extracellular ligands such as GM-CSF, tumor necrosis factor-α (TNF-α), interleukin-8 (IL-8), and CCL2 produced by immune cells and tissue structural cells [9]–[11]. However, the dysregulation of constitutive neutrophil apoptosis induces persistent neutrophil survival, which triggers or aggravates inflammatory diseases such as asthma [12], [13]. Leukocytes increase in nasal secretion following allergen exposure, and the major leukocytes are eosinophils and neutrophils [14], [15]. Although eosinophils and asthma have been widely studied by many research groups, there is little information regarding neutrophils in AR.

## Materials and Methods

### Reagents

RPMI 1640 and fetal bovine serum (FBS) were purchased from Life Technologies Inc. (Gaithersburg, MD). CLI-095, an inhibitor of Toll-like receptor (TLR) 4 (TLR4i) was purchased from Invivogen (San Diego, CA, USA). PKC δ inhibitor (rottlerin) MEK inhibitor (PD98059), and NF-κB inhibitor (BAY-11-7085) were purchased from Calbiochem (San Diego, CA, USA). GM-CSF was purchased from R&D Systems (Minneapolis, MN, USA). FITC-conjugated rabbit anti-mouse IgG, and FITC-conjugated rat anti-rabbit IgG were purchased from Molecular Probes (Eugene, OR, USA). Antibodies against phospho-tyrosine and phospho-ERK1/2 were purchased from Cell Signaling Technology (Beverly, MA, USA). Antibodies against ERK2, procaspase 3, and procaspase 9 were obtained from Santa Cruz Biotechnology (Santa Cruz, CA, USA). Anti-TLR2 antibodies were obtained from Biolegend (San Diego, CA, USA). The protease inhibitors aprotinin and E64 were obtained from Sigma–Aldrich (St. Louis, MO, USA). DP and DF were obtained from the Korea National Arthropods of Medical Importance Resource Bank (Yonsei University, Seoul, Korea) and Cosmo Bio (Tokyo, Japan). Der p1 and Der p2 were purchased from INDOOR biotechnologies (Charlottesville, VA, USA).

### Normal subjects and AR patients

Twenty-eight AR patients between 8 and 17 years of age (average  = 12.7 years) were recruited from the Department of Pediatrics at Eulji University Hospital. AR patients had general nasal symptoms for more than 4 days a week during more than 4 consecutive weeks. Allergic status was based on the presence of positive results of a skin prick test (≥2+) or multiple allergen simultaneous test (MAST) (≥ class 2) to common allergens. AR subjects suffering from asthma had mild to severe symptoms of the disease. Seventeen normal subjects between the ages of 16 and 20 years (average age  = 16.8 years) were recruited as controls. The normal subjects had no history of allergic diseases such as AR, asthma, and atopic dermatitis and did not require medication. This study was approved by the Institutional Review Board of Eulji University for normal volunteers and by the Institutional Review Board of Eulji University Hospital for AR patients. Written informed consent was obtained from study participants and from parents of children in this study. In addition, we met all the participants, informed them about the purpose of our study, and answered their questions about the project.

### Measurement of total IgE

Serum total IgE concentration was analyzed using an ADVIA Centaur immunoassay (Siemens Medical Solutions Diagnostics, Erfurt, Germany). The ADVIA Centaur IgE assay is a reverse sandwich immunoassay that employs direct chemiluminescent technology. In this system, the monoclonal mouse anti-human IgE antibodies are covalently bound to paramagnetic particles (PMP) in a solid phase. The anti-IgE coupled to the PMP captures the sample IgE, after which biotinylated allergen is added in excess and binds to the allergens-IgE antibody captured on the solid phase. Streptavidin labeled with acridinium ester is then added and binds to the biotin-labeled allergen. A direct relationship has been shown to exist between the amount of allergen-IgE present in the sample and the amount of relative light units emitted by the acridinium ester and detected by the apparatus.

### Differential count

Blood samples were obtained by venipuncture and analyzed in duplicate on an ADVIA 2120 hematology system (Siemens Medical Solutions Diagnostics). The results for all samples were verified by microscopic analysis of a blood smear.

### Neutrophil isolation and cell culture

Human neutrophils were isolated from the heparinized peripheral blood of healthy persons and AR subjects using Ficoll-Hypaque gradient centrifugation and a CD16 microbeads magnetic cell sorting kit (Miltenyi Biotec, Bergisch Gladbach, Germany). The cells were washed after hypotonic lysis to remove erythrocytes and then resuspended at 3×10^6^/ml in RPMI 1640 medium with 1% penicillin-streptomycin and 10% FBS. This method routinely yielded greater than 97% neutrophil purity as assessed by counting the cells using a cytospin system.

### Detection of apoptosis

An annexin V–fluorescein isothiocyanate (FITC) apoptosis detection kit (BD Biosciences, San Diego, CA, USA) was used for detection of neutrophil apoptosis. Isolated neutrophils were incubated with FITC-labeled annexin V and propidium iodide (PI) for 15 min at room temperature. Apoptotic neutrophils were analyzed using a FACSCalibur flow cytometer with the CellQuest software (BD bioscience) and reported as the percentage of cells showing annexin V+/PI- and annexin V+/PI+.

### Western blotting

After being treated with DP, cells were harvested and lysed in a cytosolic extraction buffer. The homogenate was then centrifuged at 10,000 g for 1 min at 4°C, after which the supernatant was collected as a cytosolic fraction. The pellet was subsequently resuspended in 50 µl of nuclear extraction buffer and centrifuged at 12,000 g for 15 min at 4°C, after which the supernatant was collected as the nuclear fraction. The protein samples (50 µg/lane) were separated by SDS-polyacrylamide gel electrophoresis, after which they were transferred to membranes and incubated with anti-phospho ERK, anti-procaspase 3 or anti-procaspase 9 antibodies and developed using the enhanced chemiluminescence detection system (Amersham Pharmacia Biotech). The same blot was stripped and reprobed with anti-β-actin or anti-ERK2 antibodies for use as an internal control.

### Immunoprecipitation

After neutrophils were incubated with DP or medium at 37°C, the cells was lysed in lysis buffer (20 mM Tris HCl, pH 7.5, 150 mM NaCl, 1 mM EDTA, 1 mM EGTA, 1% Triton X-100, 2.5 mM sodium pyrophosphate, 1 mM β-glycerol phosphate, and 1 tablet of protease inhibitor). Following incubation on ice for 20 minutes, the detergent-insoluble materials were sedimented by centrifugation at 12,000 g for 15 min at 4°C. The supernatants were then pre-cleared by incubation with 10 µl of Protein A/G Plus agarose (Santa Cruz Biotechnology). For immunoprecipitation, samples were incubated with 1 µg/ml of anti-PKCδ antibodies (Santa Cruz Biotechnology) for 2 hrs at 4°C, followed by incubation with 20 µl of Protein A/G Plus agarose for 1 hr at 4°C. The immunoprecipitates were then washed three times with ice-cold lysis buffer and boiled with 20 µl of Laemmli buffer for 4 minutes, after which Western blotting was performed using anti-phospho-PKCδ or anti-PKCδ antibodies.

### NF-κB p65 transcription factor assay

The DNA-binding activity of NF-κB was assessed using EZ-Detect transcription factor kits for NF-κB p65 (PIERCE, Rockford, IL), following the manufacturer's instructions. DNA binding specificity was assessed using wild type or mutant NF-κB oligonucleotides. chemiluminescent detection was performed using a luminometer.

### Enzyme-linked immunosorbent assay (ELISA)

The concentrations of IL-6, IL-8, GM-CSF, G-CSF, TNF-α, and CCL2 in cell supernatant were measured with a sandwich enzyme-linked immunosorbent assay (ELISA) using OptEIA Set human IL-6, IL-8, GM-CSF, TNF-α, and CCL2 (BD Biosciences, San Diego, CA, USA) and G-CSF (Abfrontier, Seoul, Korea) according to the manufacturer's instructions.

### Statistical analysis

Data were expressed as the means ± SD. Statistical differences were analyzed using a paired t-test for two-group comparisons and one-way ANOVA for comparison of more than two groups. All analyses were conducted using the SPSS statistical software package (Version 10.0, Chicago, IL), and a *p* value <0.05 was considered to indicate statistical significance.

## Results

### Exposure of HDM is closely associated with AR

**Figure 1:**
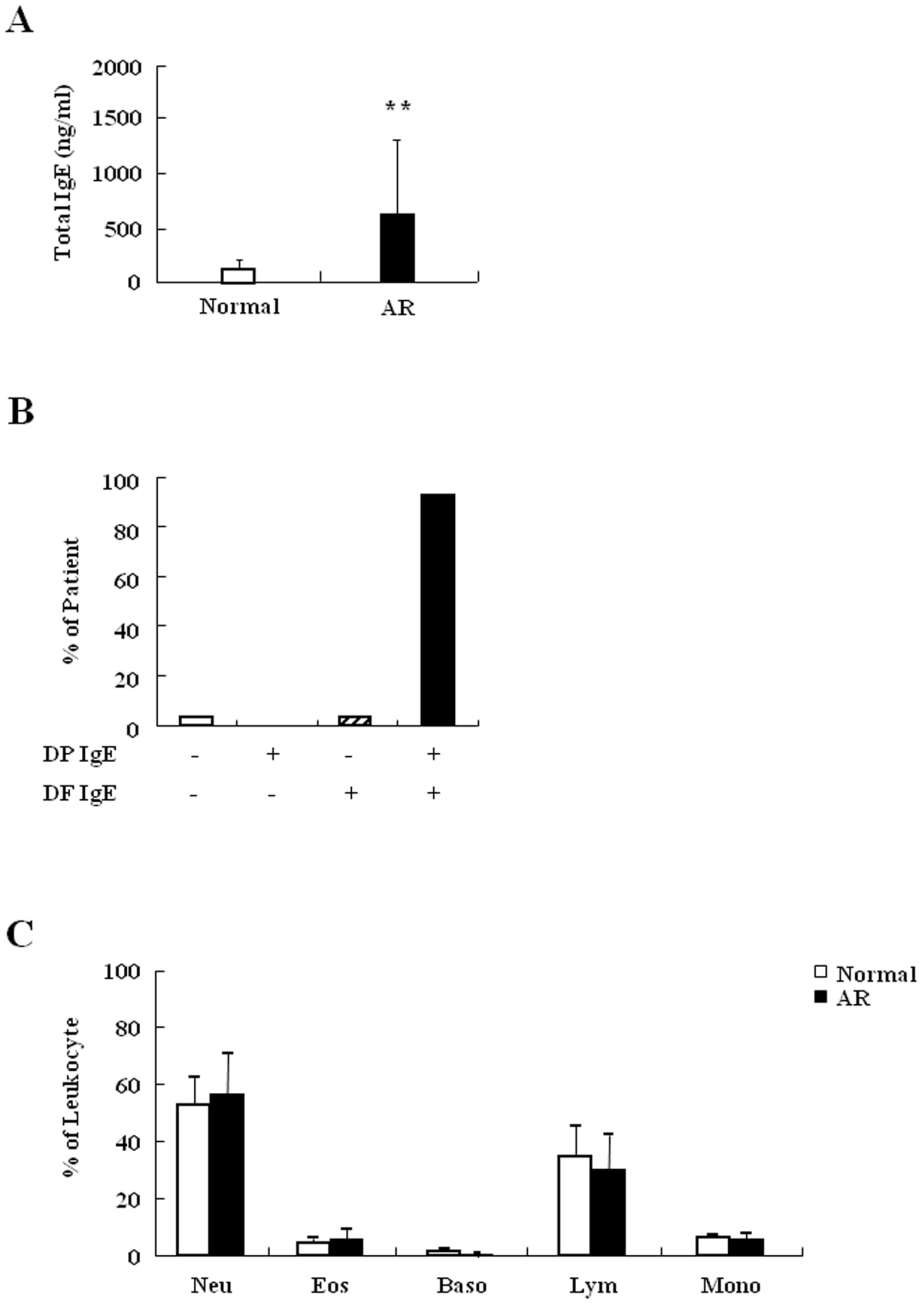
Exposure of HDM is closely associated with AR subjects. (A) Total IgE in serum of normal (n = 17) and AR subjects (n = 28) was measured by ADVIA Centaur immunoassay. (B) Allergic patients were grouped by absence or presence of DP IgE and DF IgE. The specific IgE in serum of normal and AR subjects was measured by skin prick test or MAST. (C) Blood samples were obtained by venipuncture and analyzed in duplicate on the ADVIA 2120. Neutrophils (Neu), Eosinophils (Eos), Basophils (Baso), Lymphocytes (Lym), Monocytes (Mono).

Prior to evaluating the effects of HDM in neutrophil apoptosis of normal and AR subjects, we investigated the association of HDM with AR. Exposure to allergens was detected by evaluating total IgE and allergen-specific IgE. Total IgE was significantly increased in the serum of AR subjects when compared to normal serum (Figure 1A). As shown in Figure 1B, with the exception of one patient, AR subjects recruited in this study were HDM IgE positive (+). Most HDM-specific IgE+ AR subjects were DP IgE+ and DF IgE+ (n = 26, 96.2%), and there was one DF-specific IgE+ patient. Additionally, some DP IgE+ and DF IgE+ subjects (34.6%) were crab, shrimp, cockroach, cat, or dog-specific IgE+ (Figure S1). The number of leukocytes including neutrophils and eosinophils in AR subjects was not different from that in normal subjects (Figure 1C). Taken together, these results indicate that exposure to HDM is an important cause of AR pathogenesis.

### DP inhibits constitutive apoptosis of normal neutrophils

**Figure 2:**
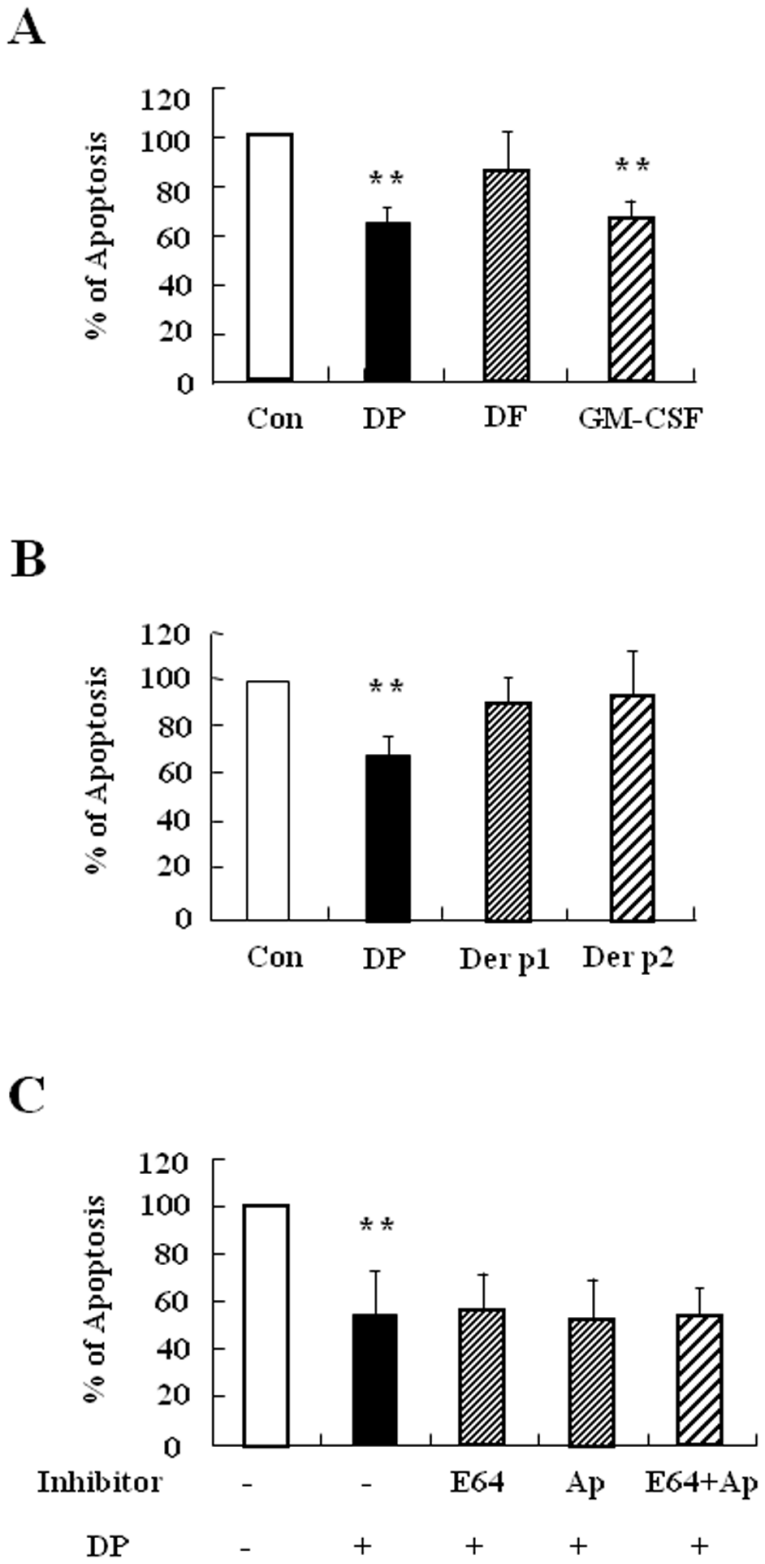
DP inhibits constitutive apoptosis of normal neutrophils. (A–C) Neutrophils were isolated from normal peripheral blood (n = 5) and then incubated for 24 h in the absence (Con) and presence of DP (10 µg/ml), DF (10 µg/ml) and GM-CSF (100 ng/ml) (A) or with and without DP (10 µg/ml), Der p 1 (10 µg/ml) and Der p 2 (10 µg/ml) (B). Neutrophils were pre-treated in the absence (Con) and presence of E64 (50 µg/ml) and aprotinin (Ap) (50 µg/ml) for 1 h, after which the cells were incubated for 24 h in the absence (Con) and presence of DP (10 µg/ml)(C). Neutrophils apoptosis was analyzed by measuring the binding of annexin V-FITC and PI. Data are presented relative to the control, which was set at 100% as the means ± SD. **p<0.01 indicates a significant difference between the control and DP-treated groups.

We examined whether DP and DF alter the regulation of neutrophil apoptosis in normal subjects. DP significantly suppressed the constitutive apoptosis of normal neutrophils (*p*<0.01), whereas DF had no effect on apoptosis (Figure 2A). To elucidate the allergen protein of DP, we examined the effects of two main proteins associated with DP allergy, Der p 1 and Der p 2. Neither Der p 1 nor Der p 2 had an effect on neutrophil apoptosis (Figure 2B). Because protease is important in HDM-induced immune regulation, we examined the relationship of protease to the anti-apoptotic effect of DP [4], [16]. E64, a cysteine protease inhibitor, and aprotinin, a serine protease inhibitor, had no impact on the inhibitory effect of DP (Figure 2C). GM-CSF was used as a positive control in this experiment. These results indicate that DP delays neutrophil apoptosis and that its mechanism may be related to a protease-independent pathway.

### DP delays neutrophil apoptosis via activation of the TLR4/PKCδ/ERK/NF-κB pathway

**Figure 3:**
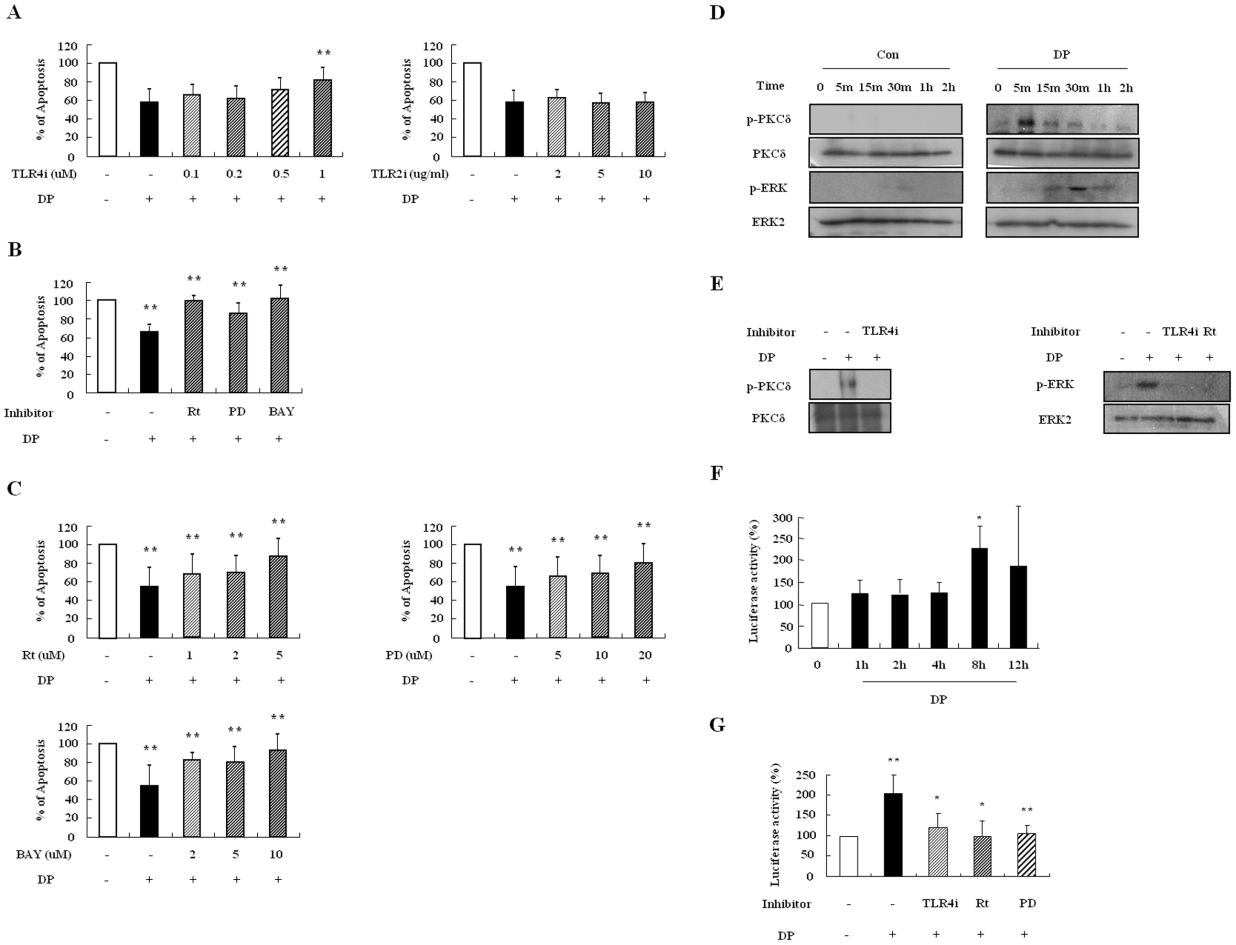
DP delays neutrophil apoptosis via activation of the TLR4/PKCδ/ERK/NF-κB pathway. (A–C) Normal neutrophils (n = 6) were pre-treated for 1 h with and without TLR4i and anti-TLR2 blocking antibodies (TLR2i) in the indicated concentration (A), with and without 5 µM rottlerin (Rt), 20 µM PD98059 (PD), and 10 µM BAY-11-7085 (BAY) (B), or with and without the indicated concentration of rottlerin (Rt), PD98059 (PD), and BAY-11-7085 (BAY) (C). Cells were incubated for 24 h in the presence and absence of DP (10 µg/ml). Apoptosis was analyzed by measuring the binding of annexin V-FITC and PI. Data are presented relative to the control, which was set at 100% and expressed as the means ± SD. **p<0.01 indicates a significant difference between the control and DP-treated groups or between the DP-treated and inhibitor-treated groups. (D) Normal blood neutrophils were incubated with DP (10 µg/ml) for the indicated time. Phosphorylation of PKCδ and ERK in the lysates was detected by Western blotting. (E) Normal blood neutrophils were pre-treated for 1 h with and without 1 µM TLR4i or 5 µM rottlerin (Rt) and incubated with DP (10 µg/ml) for 5 min or 30 min. Harvested cells were lysed, and the phospho-PKCδ (p-PKCδ) (left panel) and phospho-ERK (p-ERK) (right panel) in the lysates were detected by Western blotting. (F–G) Normal blood neutrophils were incubated with DP (10 µg/ml) for the indicated time (F). Normal blood neutrophils were pre-treated for 1 h with and without 1 µM TLR4i, 5 µM rottlerin (Rt), or 20 µM PD98059 (PD) and then incubated with DP (10 µg/ml) for 8 h (G). After harvested cells were lysed, NF-κB in the lysates was detected by luciferase assay. Data are presented relative to the control, which was set at 100% as the means ± SD. *p<0.05 and **p<0.01 indicate a significant difference between the control and DP-treated groups.

To elucidate the molecular signal mechanism involved in the delay of neutrophil apoptosis due to DP, we first examined whether DP transduces anti-apoptotic effects through TLR4. The contribution of TLR4 to the DP-induced mechanism is essential in allergic diseases [17], [18], and TLR2 is activated by DP in alveolar macrophages, which is independent or dependent on TLR4 [19]. TLR4i reversed the inhibitory effects of neutrophil apoptosis due to DP in a dose-dependent manner, and anti-TLR2 blocking antibodies had no impact on the effects (Figure 3A). To investigate downstream signaling of TLR4 due to DP, we pre-treated the samples with signal protein-specific inhibitors such as rottlerin, PD98059, and BAY-11-7085. As shown in Figure 3B and C, rottlerin, PD98059, and BAY-11-7085 suppressed the DP-induced effect in a dose-dependent manner. DP induced the phosphorylation of PKCδ and ERK, and NF-κB activity (Figure 3D and F). The phosphorylation of PKCδ and ERK was inhibited by TLR4i and rottlerin, and NF-κB activation was blocked by TLR4i, rottlerin and PD98059 (Figure 3E and G).

### DP blocks neutrophil apoptosis via suppression of the caspase9/3 pathway

**Figure 4:**
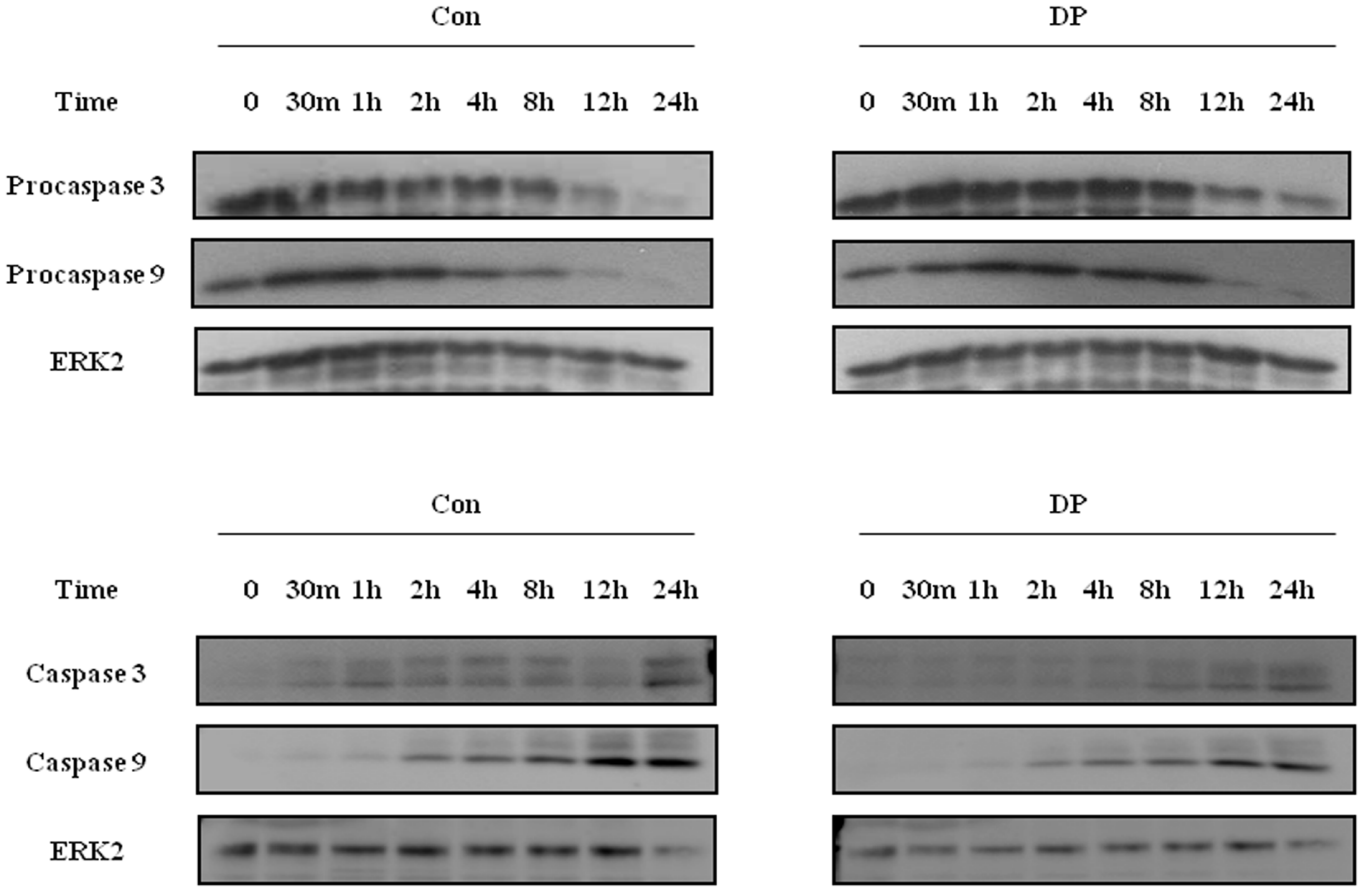
DP blocks neutrophil apoptosis via suppression of the caspase9/3 pathway. Normal blood neutrophils were incubated with 10 µg/ml of DP for the indicated time. Procaspase 3, procaspase 9 (upper panel), caspase 3 and caspase 9 (lower panel) were detected by Western blotting. The membrane was stripped and reprobed with anti-ERK2 antibodies as an internal control.

Since anti-apoptotic effects can be induced by activating the survival signal and inhibiting the pro-apoptotic signal, we investigated the alteration of procaspase 9 and procaspase 3. After beginning constitutive apoptosis, the expression of procaspase 9 and procaspase 3 decreased, and both caspases were cleaved and activated. DP delayed the time-dependent activation of caspase 9 and caspase 3 (Figure 4).

### DP triggers the suppression of constitutive apoptosis of AR neutrophils through activation of the TLR4/PKCδ/ERK/NF-κB pathway and via the suppression of caspase9/3

**Figure 5:**
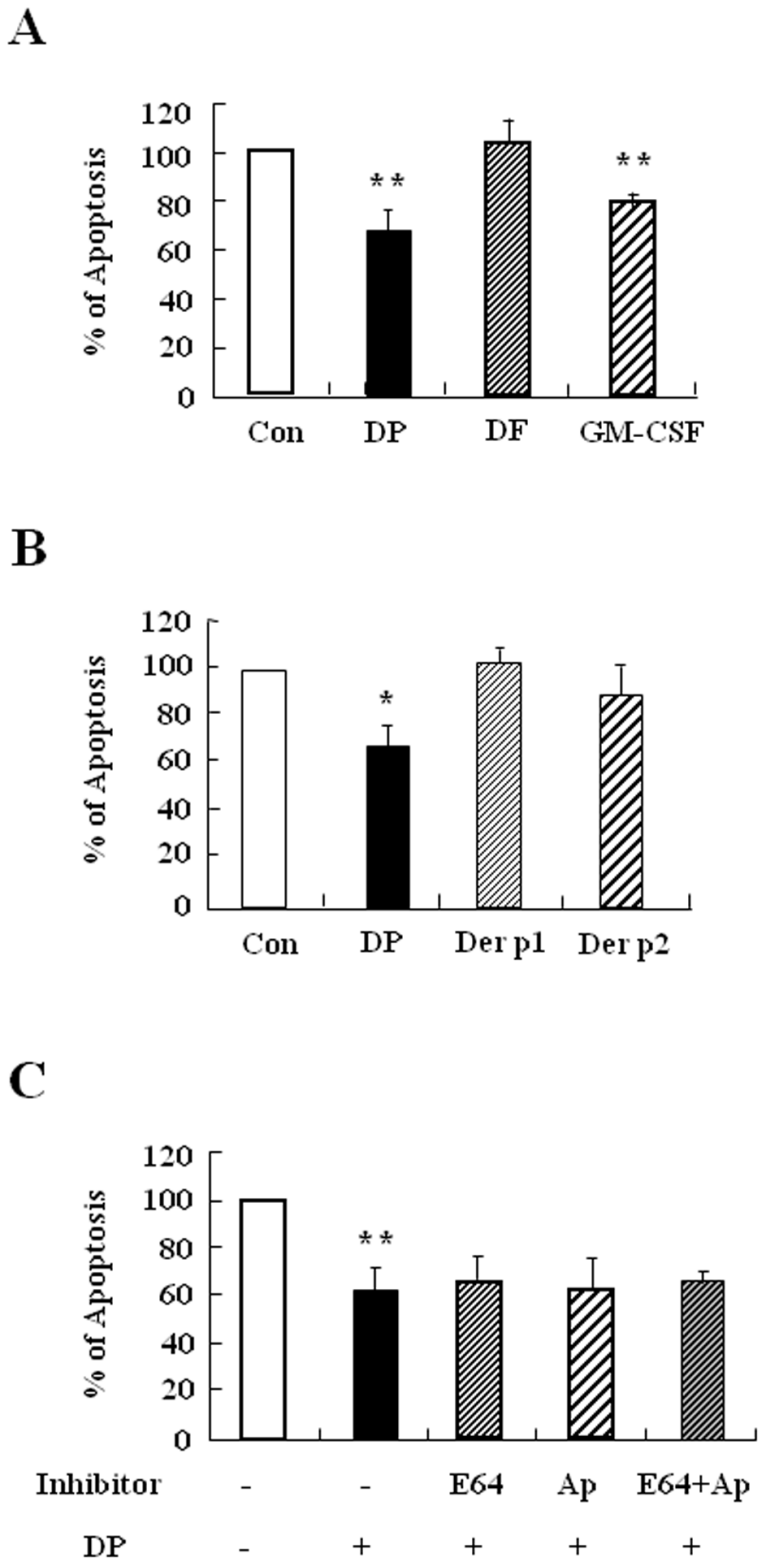
DP inhibits constitutive apoptosis of AR neutrophils. (A–C) Neutrophils were isolated from peripheral blood of AR subjects (at least n = 4) and then incubated for 24 h in the absence (Con) and presence of DP (10 µg/ml), DF (10 µg/ml) and GM-CSF (100 ng/ml) (A) or with and without DP (10 µg/ml), Der p 1 (10 µg/ml) and Der p 2 (10 µg/ml) (B). Neutrophils were pretreated in the absence (Con) and presence of E64 (50 µg/ml) and aprotinin (Ap) (50 µg/ml) for 1 h, after which the cells were incubated for 24 h in the absence (Con) and presence of DP (10 µg/ml) (C). Neutrophils apoptosis was analyzed by measuring the binding of annexin V-FITC and PI. Data are presented relative to the control, which was set at 100% as the means ± SD. *p<0.05 and **p<0.01 indicate a significant difference between the control and DP-treated groups.

**Figure 6:**
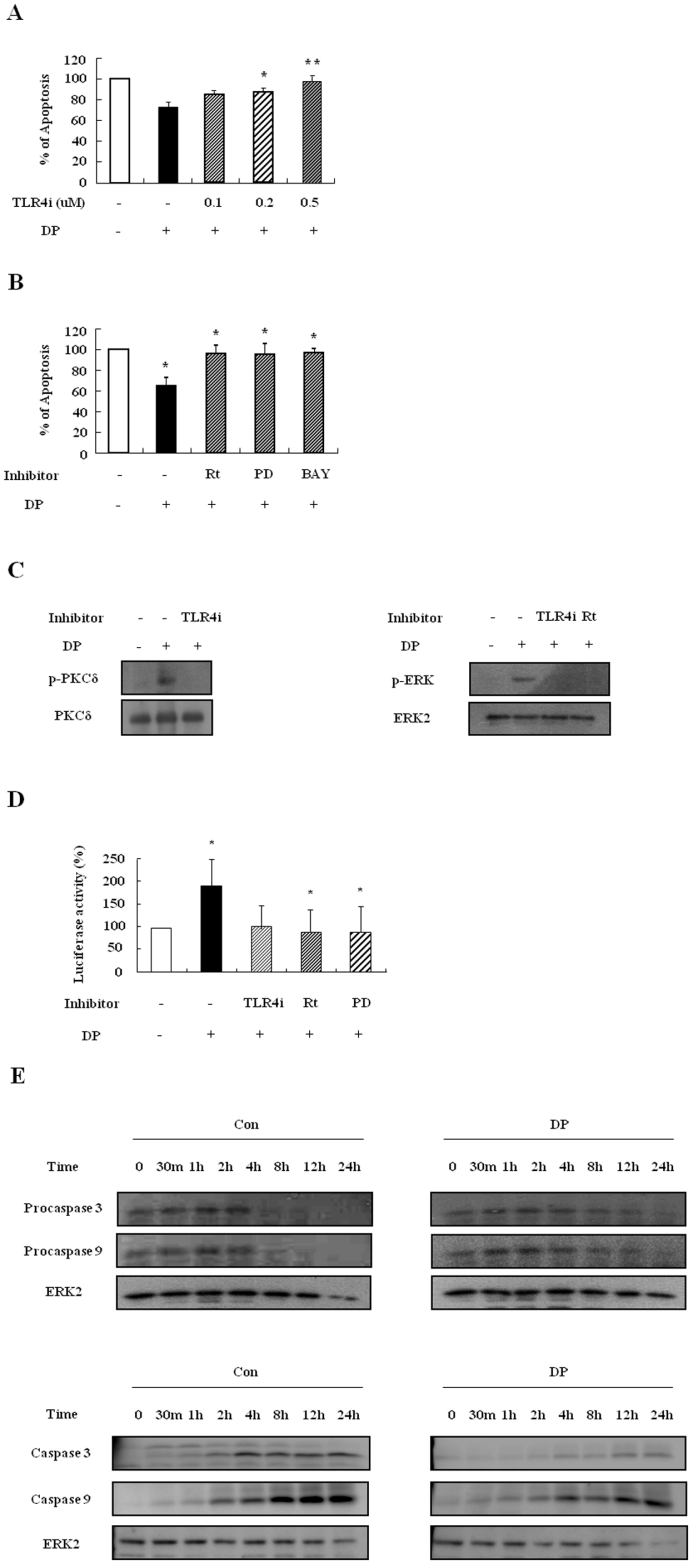
DP triggers suppression of constitutive apoptosis of AR neutrophils via activation of the TLR4/PKCδ/ERK/NF-κB pathway. (A–B) AR neutrophils (at least n = 3) were pre-treated for 1 h with and without TLR4i in the indicated concentration (A), with and without 5 µM rottlerin (Rt), 20 µM PD98059 (PD), and 10 µM BAY-11-7085 (BAY) (B. Cells were incubated for 24 h in the presence and absence of DP (10 µg/ml). Apoptosis was analyzed by measuring the binding of annexin V-FITC and PI. Data are presented relative to the control, which was set at 100% as the means ± SD. *p<0.05 and **p<0.01 indicate a significant difference between the control and DP-treated groups or between the DP-treated and inhibitor-treated groups. (C) AR blood neutrophils were pre-treated for 1 h with and without 1 µM TLR4i or 5 µM rottlerin (Rt) and incubated with DP (10 µg/ml) for 5 min or 30 min. Harvested cells were lysed, and the phospho-PKCδ (p-PKCδ) (left panel) and phospho-ERK (p-ERK) (right panel) in the lysates were detected by Western blotting. (D) AR blood neutrophils were pre-treated for 1 h with and without 1 µM TLR4i, 5 µM rottlerin (Rt), or 20 µM PD98059 (PD) and then incubated with DP (10 µg/ml) for 8 h. After harvested cells were lysed, NF-κB in the lysates was detected by luciferase assay. Data are presented relative to the control, which was set at 100% as the means ± SD. *p<0.05 indicates a significant difference between the control and DP-treated groups or between the DP-treated and inhibitor-treated groups. (E) AR blood neutrophils were incubated with 10 µg/ml of DP for the indicated time. Procaspase 3, procaspase 9 (upper panel), caspase 3 and caspase 9 (lower panel) were detected by Western blotting. The membrane was stripped and reprobed with anti-ERK2 antibodies as an internal control.

Since DP exerts an anti-apoptotic effect on normal neutrophils, we examined whether DP inhibits apoptosis of neutrophils isolated from AR subjects. As shown in Figure 5, DP blocked apoptosis of AR neutrophils, and the inhibition rate of neutrophil apoptosis of AR subjects (32%) was similar to that of normal (34%). DF did not alter the apoptosis of AR neutrophils or normal neutrophils. Der p 1, Der p 2, and cysteine and serine protease inhibitors also had no effect on apoptosis of AR neutrophils. The suppressive effect of DP was blocked by TLR4, PKCδ, ERK, and NF-κB inhibitors (Figure 6A and B). Both PKCδ and ERK were phosphorylated by DP at 5 min and 30 min, respectively. The phosphorylation of PKCδ was inhibited by TLR4i and the activation of ERK was suppressed by TLR4i and rottlerin (Figure 6C). DP induced NF-κB activation and the activity was suppressed by TLR4, PKCδ, and ERK inhibitors (Figure 6D). DP also lowered the expression of procaspase 9 and procaspase 3, indicating that it inhibited the activation of procaspase (Figure 6E). These results indicate that DP induces the suppression of neutrophil apoptosis in AR subjects, and that its mechanism is involved in the TLR4/PKCδ/ERK/NF-κB pathway.

### Inhibition of neutrophil apoptosis by DP in normal and AR subjects is associated with molecules released by DP

**Figure 7:**
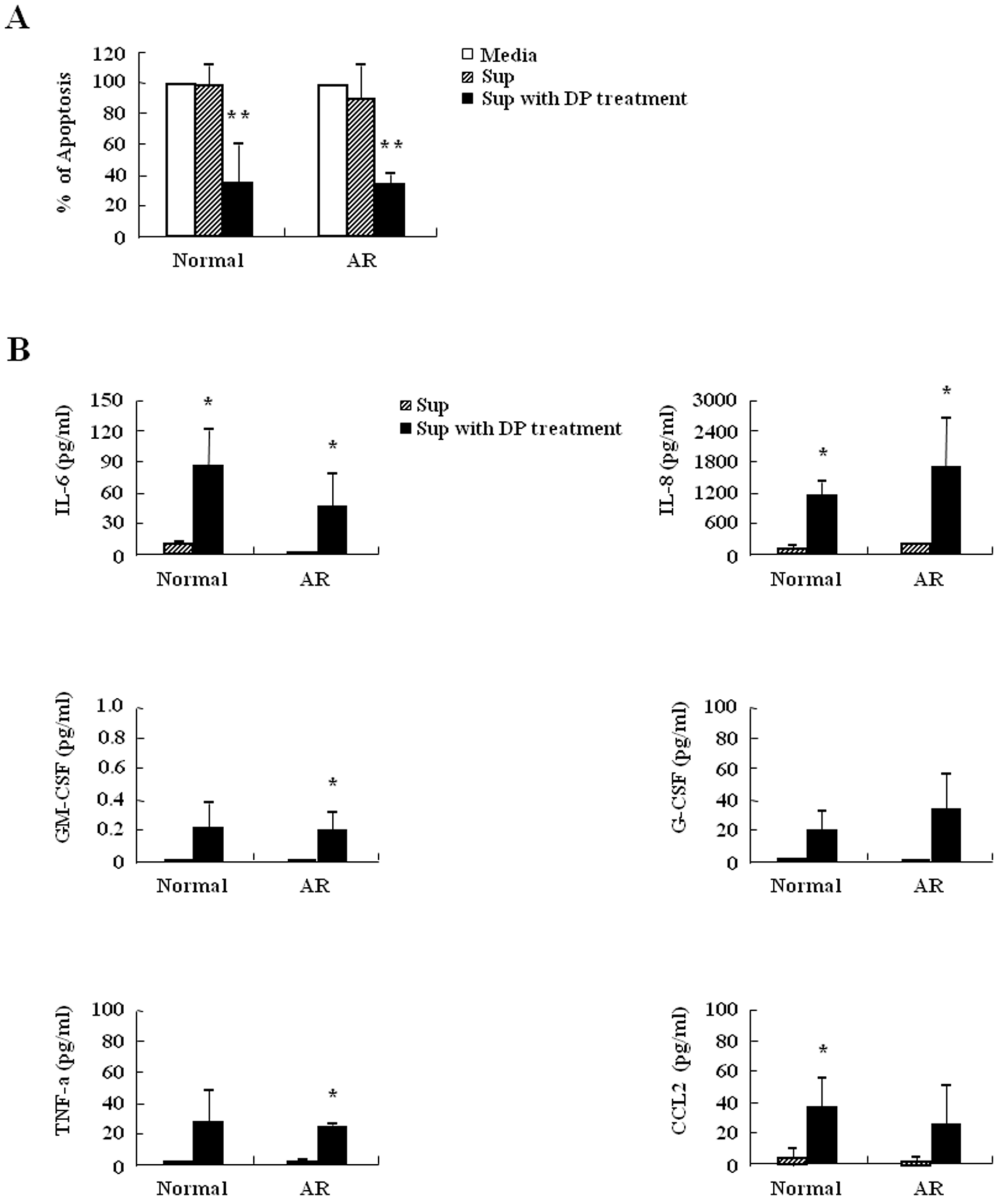
Inhibition of neutrophil apoptosis by DP is associated with molecules released by DP. (A) Neutrophils from peripheral blood of normal and AR subjects (n = 3) were incubated with or without 10 µg/ml of DP for 24 h. The supernatant (Sup) was collected and added to the fresh neutrophils obtained from the peripheral blood of normal and AR subjects. Apoptosis was analyzed by measuring the binding of annexin V-FITC and PI. Data are presented relative to the media, which was set at 100% as the means ± SD. **p<0.01 indicates a significant difference among three groups. (B) Neutrophils from peripheral blood of normal and AR subjects (n = 3) were incubated with or without 10 µg/ml of DP for 24 h. The supernatant (Sup) was collected and analyzed by ELISA. Data are expressed as the means ± SD. *p<0.05 indicates a significant difference between the superantant and the supernatant with DP treatment groups.

We examined the released factors because secretion by extracellular ligands is a meaningful process in regulation of neutrophil apoptosis. The supernatant collected from the cells after DP treatment suppressed the apoptosis of normal and AR neutrophils relative to control supernatant (Figure 7A). Therefore, we examined the survival factors of neutrophils in the supernatant. The expression of IL-6, IL-8, TNF-α, G-CSF, GM-CSF, and CCL2 increased after DP treatment in normal and AR neutrophils and this increase was not different between normal and AR subjects (Figure 7B).

## Discussion

HDM is an essential outdoor allergen that induces pathogenesis of allergic diseases. Exposure to HDM activates antigen presenting cells, which then induces production of HDM IgE in B cells. HDM-IgE is a very important diagnostic marker in atopic diseases and IgE modulates neutrophil survival [2], [20]. We observed increased total IgE in AR subjects, and most allergen-specific IgE is HDM IgE (Figure 1 and Figure S1). Few AR subjects have only DP or DF IgE, possibly owing to the immunological cross-reactivity of DP and DF, even though DP and DF have 15–20% amino acid sequence disparity [4], [21]. This phenomenon was also observed in asthmatic subjects in our previous studies [22]. However, we unexpectedly found that the effects of DP and DF on normal neutrophil apoptosis differed, and this difference was also present in AR subjects (Figure 2A and 5A). DP is important in neutrophil survival as well as atopy induction. The above results led us to investigate the exact proteins that comprise DP, which were found to be Der p 1 and Der p 2. Children presenting at an allergy clinic have high IgE recognizing Der p 1 and Der p 2, and allergic adults also have Der p 1 and Der p 2-specific IgE. These proteins activate important physiological action in allergic diseases [23], [24], but neither have an effect on neutrophil apoptosis in normal and pediatric AR patients (Figure 2B and C) (Figure 5B and C). However, further study is needed to elucidate the exact molecules of DP.

Although neutrophils in allergic disease such as asthma are involved in the severe clinical stage and in resistance to corticosteroids [25], few studies have investigated the relationship of neutrophils to AR pathogenesis. As shown in Figure 2A and 5A, DP blocked apoptosis of neutrophils. A recent study demonstrated that the number of neutrophils increases in delayed nasal response to allergen, and the authors suggested a possible association of cell-mediated hypersensitivity in the delayed nasal response [26]. Because neutrophil apoptosis is important to the resolution of inflammation, it is likely that the delay of neutrophil apoptosis due to DP increases the duration of inflammatory response, which may result in tissue damage. Our results support the suggestion that the anti-apoptotic effect of DP is associated with a protease-independent pathway (Figure 2C and 5C). TLR4 is an essential receptor to HDM in airway structural cells, and HDM activates TLR2 as well as TLR4 in macrophages [19], [27]. These studies indicate that TLR4 is important to HDM and allergic diseases. As shown in Figure 3A, the anti-apoptotic effect of DP was inhibited by TLR4 inhibitor, but not by TLR2 blocking antibodies. The anti-apoptotic downstream mechanism that occurs via TLR4 activated by DP is involved in the PKCδ/ERK/NF-κB pathway (Figure 3 and 6). Ekman et al. demonstrated that LPS activates the PKCδ/p38 MAPK/NF-κB pathway via TLR4 in nasal neutrophils and Kilpatrick et al. reported that TNF-α suppresses neutrophil apoptosis via the PKCδ/ERK pathway [9], [28], [29]. These reports indicate that anti-apoptotic signaling molecules associated with DP are important to anti-apoptotic signaling in response to other stimulators in neutrophils.

AR is an upper airway inflammatory disorder associated with allergen-specific IgE. Asthma is a lower airway allergic disease characterized by airflow obstruction [1], [30]. The inflammation pattern induced by allergen challenge is similar in the upper and lower airways. Overall, 59% of children with asthma suffer from AR [31]. AR subjects that participated in this study were divided into AR (n = 10) and AR/bronchial asthma (BA) groups (n = 18). HDM triggers differential TLR-mediated pathways between the upper and lower airways, and epithelial gene expression differs in AR and AR/BA groups [32], [33]. However, both AR and AR/BA groups showed a similar anti-apoptotic effect of neutrophils in response to DP. In addition, total IgE, HDM IgE, and leukocyte counts did not differ between the AR and AR/BA groups (Figure S2).

**Figure 8:**
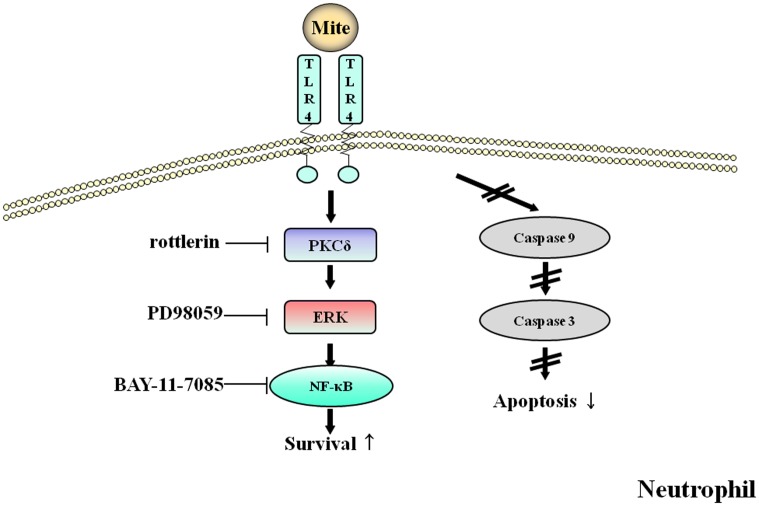
The proposed anti-apoptotic signaling pathway induced by DP in normal and AR neutrophils. Anti-apoptotic signaling due to DP involves the activation of PKCδ, ERK and NF-κB and is associated with suppression of procaspase 3 and procaspase 9 cleavage.

In summary, DP delayed constitutive apoptosis in neutrophils of normal and AR subjects and its mechanism was involved in the TLR4/PKCδ/ERK/NF-κB cascade and caspase 9/3 pathway (Figure 8). Secretion molecules are also associated with anti-apoptotic effects due to DP. These results may contribute to a better understanding of the HDM-induced allergic pathogenesis and potential approach to the treatment of allergies, including AR.

## Supporting Information

Figure S1 **Classification of AR subjects depending on allergen-specific IgE.** AR patients were grouped by allergen-specific IgE measured by skin prick test or MAST.
(TIF)

Figure S2 **Neutrophil apoptosis and IgE are not different between AR and AR/BA subjects.** (A) Neutrophils were isolated from peripheral blood of AR (n = 10) and AR/BA subjects (n = 18). The cells were incubated for 24 h in the absence (Con) and presence of 10 µg/ml of DP. Data are presented relative to the control, which was set at 100% as the means ± SD. (B) Total IgE in serum of AR (n = 10) and AR/BA subjects (n = 18) was measured by ADVIA Centaur immunoassay (C) AR (n = 10) and AR/BA subjects (n = 18) were grouped by absence or presence of DP IgE and DF IgE measured by skin prick test or MAST.
(TIF)

Figure S3 **Differential leukocyte count is not different between AR and AR/BA subjects.** Blood samples were obtained by venipuncture and were analyzed in duplicate on the ADVIA 2120. Total cells were set at 100% and data are expressed as the means ± SD.
(TIF)
